# Optical and Vibrational Spectra of CsCl-Enriched GeS_2_-Ga_2_S_3_ Glasses

**DOI:** 10.1186/s11671-016-1350-8

**Published:** 2016-03-09

**Authors:** Halyna Klym, Ivan Karbovnyk, Mariangela Cestelli Guidi, Oleksandra Hotra, Anatoli I. Popov

**Affiliations:** Lviv Polytechnic National University, 12 Bandery str., Lviv, 79013 Ukraine; Department of Electronics, Ivan Franko National University of Lviv, 107 Tarnavskogo str., Lviv, 79017 Ukraine; INFN-Laboratori Nazionali di Frascati, Via E. Fermi 40, 00044 Frascati, Italy; Lublin University of Technology, 38A ul. Nadbystrzycka, 20-618 Lublin, Poland; Institute for Solid State Physics, University of Latvia, Kengaraga 8, LV-1063 Riga, Latvia

**Keywords:** Chalcogenide, Chalcohalide glass, Optical spectra, Vibrational properties, Modification, 61.43.Fs, 71.23.Cq, 81.70Pg, 82.56Ub, 78.70.Bj

## Abstract

Optical and FTIR spectroscopy was employed to study the properties of 80GeS_2_-20Ga_2_S_3_-CsCl chalcohalide glasses with CsCl additives in a temperature range of 77–293 K. It is shown that CsCl content results in the shift of fundamental absorption edge in the visible region. Vibrational bands in FTIR spectra of (80GeS_2_-20Ga_2_S_3_)_100 − *х*_(СsCl)_*x*_ (*x* = 5, 10, and 15) are identified near 2500 cm^−1^, 3700 cm^−1^,_,_ around 1580 cm^−1^, and a feature at 1100 cm^−1^. Low energy shifts of vibrational frequencies in glasses with a higher amount of CsCl can be caused by possible thermal expansion of the lattice and nanovoid agglomeration formed by CsCl additives in the inner structure of the Ge-Ga-S glass.

## Background

Modern IR photonics emphasizes a significant importance of glassy functional materials with improved exploitation characteristics [1–3]. Among the promising media for applications of photonics are specific glasses, such as non-oxide glassy-like materials with a high content of chalcogens (S, Se, Te), which are also widely known as the chalcogenide glasses (ChGs) [4, 5]. Main developments concerning the preparation of such materials include different methods of their technological and post-technological structural modification using external influences, such as thermal annealing, high-energy irradiation, and laser beam treatment [5–8]. Technical possibilities of these modification methods, however, are, to a large extent, restricted by peculiarities of a vitreous state with characteristic effects of natural physical aging, functional non-reproducibility, and thermodynamic instability in view of high affinity to chemical reactivity.

That is why the commonly used optimization of ChG is connected with traditional chemical compositional modification based on doping possibilities with additional components introduced into the glass matrix to attain new, sometimes unusual, properties. The principal functionality of ChG is determined by their excellent IR transparency. A wide range including both commercially important atmospheric telecommunication windows at 3–5 and 8–12 μm up to a space telecommunication domain at 20–25 μm can be effectively combined with the transparency of halide compounds in a visible range by developing mixed chalcogenide-halogenide glasses such as Ge-Ga-S-CsCl systems [9, 10]. The mix of unique optical properties with high flexibility in composition and fabrication methodology makes these ChG systems compelling for IR photonics [11]. The exceptional IR transparency associated with suitable viscosity/temperature dependence creates a good opportunity for developing ChG-based molded optics for IR devices.

In [12, 13], we studied the influence of CsCl amount on an atomic-deficit sub-system (void- or pore-type structure formed due to the lack of atoms at some of glassy network sites) in Ge-Ga-S-CsCl chalcohalide composition. In this work, we analyze the CsCl effect on the optical and vibrational properties of (80GeS_2_-20Ga_2_S_3_)_100 − *x*_(CsCl)_*x*_ glasses with *x* = 5, 10, and 15.

## Methods

GeS_2_-Ga_2_S_3_-CsCl chalcogenide glasses were sintered from Ge, Ga, S, and CsCl compounds (99.999 % purity), as described in details elsewhere [14–16]. Raw materials were melted at 850 °C in a silica tube for several hours. The (80GeS_2_-20Ga_2_S_3_)_100 − *х*_(СsCl)_*x*_ (*x* = 5, 10, and 15) glasses were annealed at 15 °C below a glass transition temperature (*T*_g_) for each of the glasses [16] to minimize inner strains. Such amount of CsCl additives is optimal in order to modify Ge-Ga-S glasses before future doping of these materials by rare-earth ions. For the purpose of convenience, the obtained glasses of (80GeS_2_-20Ga_2_S_3_)_100_(СsCl)_0_, (80GeS_2_-20Ga_2_S_3_)_95_(СsCl)_5_, (80GeS_2_-20Ga_2_S_3_)_90_(СsCl)_10_, and (80GeS_2_-20Ga_2_S_3_)_85_(СsCl)_15_ hereafter are referred to as (CsCl)_0_, (CsCl)_5_, (CsCl)_10_, and (CsCl)_15_, respectively.

Optical spectra were measured using a Cary5 (Varian) short-wavelength spectrophotometer. A Bruker Vector 22 instrument was exploited to record the spectra in the mid and far-infrared regions [16]. IR spectroscopy at different temperatures was carried out at the infrared beamline SINBAD of the Daphne Light synchrotron IR facility [17–21]. The infrared transmission spectra were collected using a Vertex 70V FTIR spectrometer equipped with a Janis ST-100-FTIR (Janis Research Company, LLC, Woburn, MA) continuous flow cryostat and a room temperature DTGS detector. The outer cryostat windows were made of CaF_2_. The temperature points (293, 220, 150, and 77 K) were set with a LakeShore 331 temperature controller and kept constant, controlling the flux of liquid nitrogen and heating power during the necessary measurement procedure. The heating/cooling rate in experiments was set to 10 K min^−1^. The transmission spectra were acquired in the vacuum between 4500 and 900 cm^−1^ with a spectral resolution of 4 cm^−1^, performing 128 scans.

## Results and Discussion

**Fig. 1:**
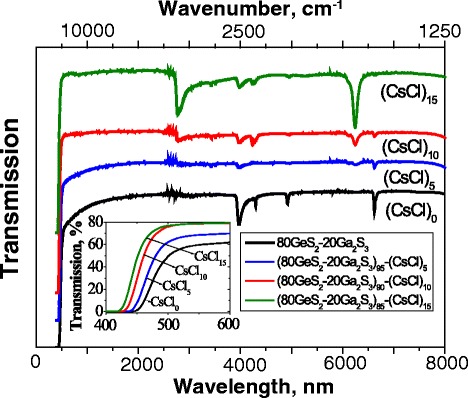
Visible and IR transmission spectra of (80GeS_2_-20Ga_2_S_3_)_100 − *х*_(СsCl)_*x*_ (*x* = 0, 5, 10, and 15) chalcohalide glasses measured at 293 K

The transmission in the visible and infrared region of spectra for ChG under study measured at 293 K is shown in Fig. 1. Samples with *x* = 0 and *x* = 5 are essentially transparent down to 500 nm. A further increase of the CsCl content in the base GeS_2_-Ga_2_S_3_ glassy matrix results in a shift of the absorption edge towards shorter wavelengths which is in line with the earlier reports [14, 16]. The transmission increases with a CsCl concentration from 60 % in CsCl_0_ to 80 % in CsCl_15_. As was shown in [16], by adding up to 15 mol% of the alkali halide in the glassy matrix, the bandgap energy evolves from 2.64 to 2.91 eV. From a structural point of view, the addition of less than 15 % of CsCl in GeS_2_-Ga_2_S_3_ glasses is characterized by the formation of GaS_4 − *x*_Cl_*x*_ tetrahedra that are dispersed in the glass network. Hence, the average number of Ga–S bonds decreases in favor of the average number of Ga–Cl bonds. One of the observed features for (80GeS_2_-20Ga_2_S_3_)_100 − *х*_(СsCl)_*x*_ (*x* = 0, 5, 10, and 15) glasses in the vis-IR transmission is the sharp peak at 4 μm (for base glass, black line) that is attributed to S–H stretching and is considerably damped for samples with CsCl. There are also features at 6300 nm corresponding to H_2_O, in which 6700 and 2900 nm are related to O–H stretching vibrations. The intensity of absorption bands associated with water increase with the CsCl amount, confirming the hygroscopicity of CsCl [16].

**Fig. 2:**
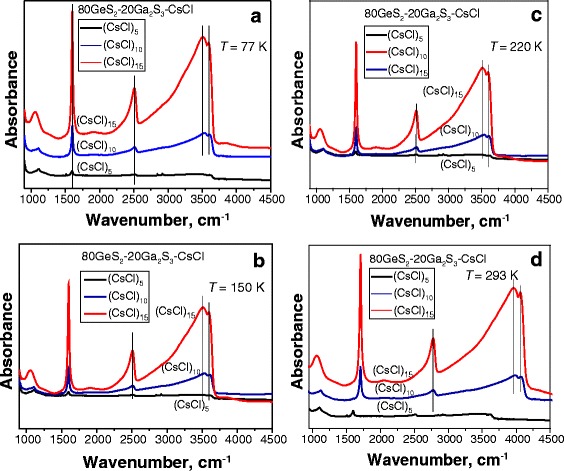
FTIR vibrational spectra for (80GeS_2_-20Ga_2_S_3_)_100 − *х*_(СsCl)_*x*_ (*x* = 5, 10, and 15) chalcohalide glasses at different CsCl contents measured at 77 K (**a**), 150 K (**b**), 220 K (**c**), and 293 K (**d**)

**Fig. 3:**
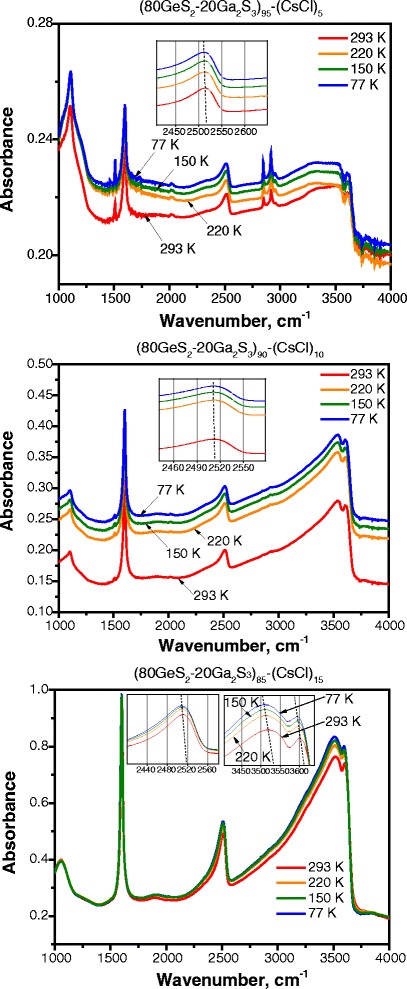
FTIR vibrational spectra for (80GeS_2_-20Ga_2_S_3_)_100 − *х*_(СsCl)_*x*_ (*x* = 5, 10, and 15) chalcohalide glasses at different temperatures

The results of FTIR measurements at different temperatures are shown in Figs. 2 and 3. First, the absorbance for GeS_2_-Ga_2_S_3_ as a function of CsCl content was analyzed at 77, 150, 220, and 293 K and is presented in Fig. 2. Three main vibrational bands are identified for (80GeS_2_-20Ga_2_S_3_)_100 − *х*_(СsCl)_*x*_ (*x* = 5, 10, and 15) glasses. The fundamental S–H absorption band is observed at 2500 cm^−1^ [22]. The maxima at ~3700 cm^−1^ are very likely an O–H-related absorption, common for many chalcogenide glasses [22]. The band at around 1580 cm^−1^ corresponds to H_2_O impurity that resides in structural voids [23]. The feature at ~1100 cm^−1^ could be due to inorganic sulfate ion absorption [24]. For each temperature, the intensities of the identified bands are increased with the CsCl amount in the 80GeS_2_-20Ga_2_S_3_ ChG. The S–H and O–H absorption bands are very weak for (80GeS_2_-20Ga_2_S_3_)_95_(СsCl)_5_ glasses. There is a minor band near 1580 cm^−1^ that corresponds to H_2_O. Obviously, the structures of Ge-Ga-S glasses containing a smaller amount of CsCl are more stable against the adsorption of water due to a small amount of free-volume voids [12, 25]. Increasing of CsCl content in (80GeS_2_-20Ga_2_S_3_)_100 − *х*_(СsCl)_*x*_ glasses to *x* = 10 and *x* = 15 results in higher intensities of the bands corresponding to 1580 and ~3700 cm^−1^ at all measured temperatures (Fig. 2). These transformations are connected with changes in the inner structure of ChG with CsCl that facilitate a more intensive H_2_O adsorption. Most probably, (CsCl)_15_ glasses could be enriched by the water molecules, having an effect on its structure. As it follows from the measurement results, main vibration bands are somewhat shifted to lower energies at 77 and 150 K, but as the temperature rises up to 220 and 293 K, these changes become more obvious. Such temperature shifts of vibrational frequencies can be caused by possible thermal expansion of the lattice [26] and nanovoid agglomeration. The fact of the nanovoid formation induced by CsCl additives in Ge-Ga-S has been definitely confirmed by positron annihilation lifetime spectroscopy [12, 15, 25, 27–29].

The vibrational spectra of (80GeS_2_-20Ga_2_S_3_)_100 − *х*_(СsCl)_*x*_ (*x* = 5, 10, and 15) glasses collected at different temperatures for each composition are shown in Fig. 3. It can be observed that the bands located near 1580 cm^−1^ appear for all glasses and show no shifts. In the (CsCl)_5_ and (CsCl)_10_ ChG samples, there is a very slight shift of the S–H absorption band located near 2500 cm^−1^ at elevating temperatures. However, the temperature shift of vibrational frequencies towards lower energies for (CsCl)_15_ glasses is detected for the S–H vibrational band and O–H-related peaks also shift with the increase of temperature from 77 to 293 K. We tend to think that this effect may be due to the differences in water absorption in the structure at higher temperatures. All transformations of vibrational modes of (80GeS_2_-20Ga_2_S_3_)_85_(СsCl)_15_ glasses are related to the excessive amount of CsCl additive, resulting in the damage to the inner structure of the host material.

## Conclusions

The CsCl addition effects on the optical and vibrational spectra for GeS_2_-Ga_2_S_3_-CsCl glasses are investigated. It is demonstrated that the transmission in the visible region increases with a CsCl concentration from 60 % in (CsCl)_0_ to 80 % in (CsCl)_10_ and (CsCl)_15_. A sharp peak at 4000 nm for a base (CsCl)_0_ glass is attributed to S–H stretching and is considerably damped for samples with CsCl in the mid-IR spectra. There are also features at 6300 nm corresponding to H_2_O, in which 6700 and 2900 nm are related to O–H stretching vibrations. The intensity of absorption bands associated with water increased with the CsCl amount, confirming the hygroscopicity of CsCl. It is established that for each measured temperature (77, 150, 220, and 293 K), the intensities of the observed vibrational bands increase with the CsCl amount in the 80GeS_2_-20Ga_2_S_3_ ChG. The principal vibrational bands centered near 1100, 1580, 2500, and 3700 cm^−1^ in (CsCl)_15_ glasses are slightly shifted to lower energies between 77 and 150 K. The shift is more pronounced between 220 and 293 K. Such temperature shifts of vibrational frequencies can be caused by possible thermal expansion of the lattice and agglomeration of nanovoids formed by CsCl additives. Temperature dependences of FTIR spectra also indicate the lower-energy shift of vibrational frequency of the S–H-related band for (CsCl)_15_ glasses and the shift of O–H-related peaks at elevating temperatures.
